# Gold Half-Shell-Coated Paclitaxel-Loaded PLGA Nanoparticles for the Targeted Chemo-Photothermal Treatment of Cancer

**DOI:** 10.3390/mi14071390

**Published:** 2023-07-08

**Authors:** Jaime Ibarra, David Encinas-Basurto, Mario Almada, Josué Juárez, Miguel Angel Valdez, Silvia Barbosa, Pablo Taboada

**Affiliations:** 1 Departamento de Física, Matemáticas e Ingeniería, Universidad de Sonora, Campus Navojoa, Navojoa 85880, Sonora, Mexico; david.encinas@unison.mx; 2 Departamento de Ciencias Químico-Biológicas y Agropecuarias, Universidad de Sonora, Campus Navojoa, Navojoa 85880, Sonora, Mexico; mario.almada@unison.mx; 3 Departamento de Física, Universidad de Sonora, Campus Hermosillo, Hermosillo 83000, Sonora, Mexico; josue.juarez@unison.mx (J.J.); miguel.valdes@unison.mx (M.A.V.); 4 Departamento de Física de Partículas, Universidad de Santiago de Compostela, 15782 Santiago de Compostela, A Coruña, Spain; silvia.barbosa@usc.es

**Keywords:** paclitaxel, PLGA, half shell, cyRGDk peptide, chemo-photothermal therapy

## Abstract

Conventional cancer therapies suffer from nonspecificity, drug resistance, and a poor bioavailability, which trigger severe side effects. To overcome these disadvantages, in this study, we designed and evaluated the in vitro potential of paclitaxel-loaded, PLGA-gold, half-shell nanoparticles (PTX-PLGA/Au-HS NPs) conjugated with cyclo(Arg-Gly-Asp-Phe-Lys) (cyRGDfk) as a targeted chemo-photothermal therapy system in HeLa and MDA-MB-231 cancer cells. A TEM analysis confirmed the successful gold half-shell structure formation. High-performance liquid chromatography showed an encapsulation efficiency of the paclitaxel inside nanoparticles of more than 90%. In the release study, an initial burst release of about 20% in the first 24 h was observed, followed by a sustained drug release for a period as long as 10 days, reaching values of about 92% and 49% for NPs with and without near infrared laser irradiation. In in vitro cell internalization studies, targeted nanoparticles showed a higher accumulation than nontargeted nanoparticles, possibly through a specific interaction of the cyRGDfk with their homologous receptors, the ανβ3 y ανβ5 integrins on the cell surface. Compared with chemotherapy or photothermal treatment alone, the combined treatment demonstrated a synergistic effect, reducing the cell viability to 23% for the HeLa cells and 31% for the MDA-MB-231 cells. Thus, our results indicate that these multifuncional nanoparticles can be considered to be a promising targeted chemo-photothermal therapy system against cancer.

## 1. Introduction

The search for new colloidal nanoparticles with a defined morphology, anisotropy, and physicochemical properties has recently increased in the last two decades [1,2]. Designing nanosized particles with multiple functions has recently emerged as a very interesting approach to treating and diagnosing chronic diseases such as cancer [3]. These multifunctional nanoparticles (MNPs) present different physical and chemical properties that are absent in traditional homogeneous nanomaterials, providing them with a set of characteristics that allow for multiple applications in the biomedical field, including drug delivery, sensing, and imaging [4,5]. For example, a single particle with multiple domains can simultaneously achieve multidrug loading and be an ideal carrier of near-infrared (NIR) resonant nanomaterials in different geometries such as nanospheres, nanorods, nanoshells, and nanostars. Gold-based nanomaterials can function as photothermal therapy agents or excellent optical tracer/contrast agents for imaging modalities such as dark-field microscopy, optical coherence tomography, photoacoustic tomography, and multiphoton luminescence detection [6].

Photothermal therapy (PTT) has emerged as a potential anticancer treatment for delivering localized cytotoxic heat to tumor cells; additionally, when combined with targeted delivery and chemotherapy, its therapeutic efficacy is enhanced significantly [7,8]. For instance, Ren et al. developed a nanosystem for combined chemotherapy and PTT using paclitaxel-loaded gold nanorods (PTX-Au NR). Their in vitro cell assay was shown to be highly effective in killing head, neck, and lung cancer cells by combining PTT and chemotherapy when the PTX-Au NRs were irradiated with low-intensity NIR light [9]. Agabeigi et al. prepared folate/methotrexate-loaded silica-coated gold nanoparticles with an excellent photothermal transfer ability upon near-infrared light irradiation. They investigated the combined effect of their system and low-level laser therapy (810 nm) against breast cancer cells, indicating that the highest antiproliferative effect on MDA-MB-231 cell lines was observed in the cells exposed to a combination of the MTX-FA-loaded Au@SiO_2_ NPs and NIR light, proving the efficacy of combination chemo-photothermal therapy [10]. In addition, Banstola et al. developed PTX-loaded PLGA microspheres coated with gold nanoparticles for pancreatic cancer treatment, observing that Panc-1 cells treatment with GNPs-pD-PTX-PLGA-Ms and NIR irradiation exhibited the highest cellular cytotoxicity, with a significantly lower viability, in comparison to a photothermal or chemotherapeutic agent alone, suggesting that their nanosystem could provide synergistic chemo-photothermal therapy for pancreatic cancer treatment [11].

Gold nanoshells, which are composed of a spherical dielectric core and thin nanoscale shell or half-shell, present high light absorption in the near-infrared (NIR) region, where light can penetrate the body’s deep tissues [7,12]. Recently, several sorts of reduced symmetrical gold nanoshells have been fabricated, such as half-shells, demonstrating that reducing the symmetry of gold nanoshells has several interesting consequences, such as highly tunable optical properties, and also renders their optical properties dependent on the angle and polarization of the incident light, exhibiting red-shifted plasmon absorption relative to simple nanospheres; this is why half-shell structures have shown the ability to locally enhance the electromagnetic field [13]. Poly(lactic-co-glycolic acid) (PLGA) as a dielectric core has been explored for designing drug delivery systems that allow for a combination of chemotherapy and localized hyperthermy. Park et al. encapsulated doxorubicin in PLGA nanoparticles coated with gold half-shells and reported a photothermally controlled drug delivery in response to NIR irradiation; moreover, the combined treatment demonstrated a synergistic effect against the HeLa cell line [14]. Lee et al. investigated the beneficial effects of targeted chemo-photothermal on multi-drug-resistant xenograft tumors in mice using a similar system as Park et al.; they exposed nanoparticle-treated tumors to near infrared light for 10 min, promoting a temperature increase to 45 °C and a significant reduction in the rate of tumor growth, using a much smaller dose of doxorubicin compared to the common dose used in traditional chemotherapy [8]. Lee et al. developed an NIR-controlled drug delivery system for methotrexate using PLGA nanoparticles coated with gold half-shells and functionalized with arginine-glycine-aspartic acid (RGD) peptides for the treatment of rheumatoid arthritis, promoting local heat and drug release enhancement upon irradiation due to the Au half-shells [15].

MNPs can be an efficient approach to treating several diseases, including cancer. A combination of photothermal and chemotherapy has shown an enhanced therapeutic effect; furthermore, PLGA nanoparticles coated with gold half-shells have demonstrated the promising controlled release of different drugs in response to laser irradiation. Herein, we report on Au half-shell PLGA nanoparticles with modified-surface moieties cyclo-RGD peptide (cyRGDfk) as an NIR-response drug delivery system for PTX. This system showed an enhanced intracellular accumulation and in vitro therapeutic effects against cancer cell lines.

## 2. Materials and Methods

### 2.1. Materials

Poly-(DL-lactide-co-glycolide) (PLGA) 50:50 with Mw 30,000–60,000 Da, poly (vinyl alcohol) (Mw 31,000–50,000 Da), acetic acid (99%), paclitaxel semi-synthetic (PTX), fluorescein isothiocyanate (>90%), acetone (99%), carbodiimide hydrochloride (EDC), and sulfo-NHS were purchased from Sigma-Aldrich Chemical Co. (St. Louis, MO, USA). Chitosan with a deacetylation degree of 79% and average molecular weight of 150 kDa was acquired from Fluka. Thiol-PEG Carboxylic acid (SH-PEG-COOH, Mn 2400 Da) was purchased from Polymer Source Inc. (Dorval, QC, Canada). The cyclic RGDfk (Cyclic Arg-Gly-Asp-D-Phe-Lys) peptide (RGD) was purchased from BACHEM AG. (Seoul, Republic of Korea). The breast MDA-MB-231 and cervical Hela cancerous cells were obtained from Biolabs cell (San Diego, CA, USA). Prolong Gold Antifade reagent with DAPI, Dulbecco’s modified eagle medium, fetal bovine serum, penicillin/streptomycin, sodium pyruvate, and MEM non-essential amino acids (NEAA) were purchased from Invitrogen (Carlsbad, CA, USA).

### 2.2. Preparation of PTX-PLGA/CS NPs

PTX-PLGA/CS NPs were prepared using the single-emulsion solvent evaporation method [16]. Briefly, 50 to 100 μg of PTX and 6 mg of PLGA were dissolved in 0.6 mL of acetone, then, the organic phase was poured dropwise into 10 mL of an aqueous phase under moderate stirring for 5 min and the resulting suspension was placed inside an extraction hood for 12 h to remove the organic phase. Once the organic phase was removed, the NP suspension was poured dropwise into an aqueous phase that contained PVA (3% *w*/*v*) and CS (3.33 × 10^−5^ M), and the preparation was stirred with a magnetic stirrer at room temperature for 4 h. After, the PTX-PLGA/CS NPs were collected via centrifugation at 9000 rpm for 45 min at 4 °C and washed twice with deionized water. Finally, the PTX-PLGA/CS NPs were stored for further characterization.

Empty NPs were obtained in the same manner, without including PTX.

### 2.3. Preparation of cyRGDfk-Conjugated PTX-PLGA/CS-Au Half-Shell NPs (PTX-PLGA/Au-HS-RGD NPs) 

Once the PTX was encapsulated within the PLGA/CS NPs, we developed the PTX-PLGA/CS-Au half-shell NPs (PTX-PLGA/CS-Au-HS NPs) and conjugated the cyRGDfk peptide to the surface of the half-shell. First, spin-coating deposited a monolayer of the PTX-PLGA/CS NPs onto a silicon substrate. Next, the PTX-PLGA/CS NPs’ faces, exposed at the surface of the substrate, were coated with an Au layer of a 10 nm thickness via physical vapor deposition. After the deposition of the Au layer, the substrates were placed inside a 0.5% *w*/*v* SH-PEG-COOH solution and COOH-terminated PTX-PLGA/Au half-shells NPs were released via sonication and collected via centrifugation. Finally, cyRGD-conjugated PTX-PLGA/Au half-shell NPs (PTX-PLGA/-Au-HS-RGD NPs) were obtained via carbodiimide chemistry through the amide bond formation between the amine group of cyRGDfk and carboxylic groups of the PTX-PLGA/Au-HS NPs activated with EDC and sulfo-NHS.

### 2.4. Characterization of PTX-PLGA/Au-HS-RGD NPs

The morphological characteristics of the nanoplatforms were examined using scanning electron microscopy (FE-SEM, S-4160, Hitachi, Japan) and transmission electron microscopy (Phillips CM-12). The size distributions and ζ potential were analyzed using dynamic light scattering (DLS, Zetasizer Nano ZS, Malvern Instruments Ltd., Malvern, UK). The absorption spectra were obtained using a Cary Bio 100 UV-vis spectrophotometer (Agilent Technologies, Santa Clara, CA, USA).

### 2.5. Determination of PTX Content in the PTX-PLGA/Au-HS-RGD NPs

The PTX contained inside the PLGA/-Au-HS-RGD NPs was determined using high-performance liquid chromatography (HPLC) [17]. For this purpose, the NP solution was centrifugated at 9000 rpm for 30 min, and 50 μL of the supernatant was carefully removed for quantification. Chromatographic analyses were performed with an HPLC 1200 system (Agilent Technologies, Santa Clara, CA, USA) using a Phenomenex Luna C 18 (30 mm × 2 mm, 5 μm particle size) reversed-phase column. The column was thermostated at 30 °C and the analysis was carried out in isocratic mode with a mobile phase comprising water (containing 0.1% acetic acid) and acetonitrile (20/80, *v*/*v*) at a 0.8 mL min^−1^ flow rate for 10 min. The PTX was quantified using UV detection at 227 nm using calibration curves linear over the range of standard concentrations at 1–40 μg mL^−1^, with a correlation coefficient of 0.998. The drug incorporation efficiency (EE%) was expressed as the percentage of the drug in the NPs to the amount used in their preparation (Equation (1)):

(1) $$EE\left(\%\right)=\frac{Amount\;of\;drug\;in\;NPs}{Initial\;amount\;of\;drug}\times 100$$

Three replicates were carried out to assess the average PTX content in the PTX-PLGA/Au-HS-RGD NPs.

### 2.6. Photothermal Properties

The NIR-laser-induced temperature increase in the PLGA/Au-HS-RGD NPs was determined using a continuous wave fiber-coupled diode laser source with an 808 nm wavelength (50 W, Oclaro Inc., San Jose, CA, USA). First, the PLGA/Au-HS-RGD NP suspensions were adjusted at optical densities of 0.2, 0.4, 0.6, 0.8, and 1. Then, 2 mL of each suspension was placed inside a quartz cuvette and irradiated at powers of 0.3, 0.5, 0.8, and 1 W cm^−2^ over 15 min, using a final diameter size of the beam spot of 0.5 cm. The temperature increases were measured every 30 s using a Spark Science Learning System model PS-2008A thermocouple. All the measurements were performed in triplicate.

### 2.7. In Vitro PTX Release

The release profile of the PTX from the PLGA/Au-HS-RGD NPs was assessed in vitro at 37 ºC in PBS buffer (pH 7.4). The release experiments were conducted with and without exposure to NIR light, with a laser diode of an 808 nm wavelength and 0.5 W cm^−2^ beam diameter (50 W, Oclaro Inc., San Jose, CA, USA). Briefly, 2 mL of the PTX-PLGA/Au-HS-RGD NPs with a known PTX concentration (25 μM) were placed in a quartz cuvette and maintained under mild constant shaking (150 rpm). The samples were NIR light irradiated for 5 min at 3, 12, and 24 h after the start of the experiment. At a predetermined time, aliquots of 0.5 mL were withdrawn from the incubation medium and centrifuged at 10,000 rpm for 10 min at 20 °C. Then, the supernatant was carefully extracted and the PTX content was determined using HPLC (above mentioned). The concentration of PTX released was expressed as a percentage of the total PTX in the NPs and plotted as a function of time. All the measurements were performed in triplicate.

### 2.8. Fluorescent Labeling of PLGA/Au-HS-RGD NPs

FITC as a fluorescent marker was loaded onto the PLGA/Au-HS-RGD NPs using the solvent evaporation method. Briefly, 0.5 mg of FITC and 6 mg of PLGA were dissolved in 0.6 mL of acetone, then the organic phase was poured dropwise into 10 mL of an aqueous phase under moderate stirring for 5 min, and the resulting suspension was placed inside an extraction hood for 12 h to remove the organic phase. Subsequently, the same protocol used for the formulation of the PLGA/Au-HS-RGD NPs was followed.

### 2.9. Cellular Uptake

The HeLa cells were seeded on poly-L-lysine-coated glass coverslips (12 × 12 mm) placed inside 6-well plates at a density of 150,000 cells/well in 3 mL of DMEM medium (supplemented with 10% of fetal serum bovine, 1% penicillin-streptomycin, 1% NEAA, and 1% sodium pyruvate) and grown to 90% confluency under standard culture conditions. Then, the medium was discarded and the cells were washed with PBS with a pH of 7.4. Subsequently, the cells were exposed to FITC-PLGA/Au-HS NPs and FITC-PLGA/Au-HS-RGD NPs contained in a 3 mL DMEM medium. After 4 h of incubation, the medium with the NPs was aspirated and the cells were fixed with paraformaldehyde 4% (*w*/*v*) for 10 min, washed with PBS (three times), permeabilized with 0.2% (*w*/*v*) Triton X-100 for 10 min, washed with PBS (three times), and stained with BODIPY^®^ Phalloidin (Invitrogen) for 30 min. Finally, the cells were washed with PBS (three times), mounted on a glass slide, stained with ProLong^®^ Gold antifade DAPI (Invitrogen), and stored for 24 h at −20 °C. The samples were visualized with 20× objective using a confocal spectral microscope Leica TCS-SP2 (Leica Microsystems GmbH, Heidelberg Mannheim, Germany).

### 2.10. In Vitro Cell Viability Assays

We performed a series of in vitro cell viability assays to evaluate the anticancer potential of the PTX-PLGA/Au-HS-RGD NPs against the HeLa and MDA-MB-231 cells using a CCK8 assay. Briefly, all the cells were seeded in 96 well plates at a density of 10,000 cells/well and cultured in Dulbecco’s modification of eagle’s medium (DMEM) (supplemented with 10% of fetal serum bovine, 1% penicillin-streptomycin, 1% non-essential amino acids, and 1% sodium pyruvate) for 24 h, under a humidified atmosphere of 5% CO_2_ at 37 °C in an incubator. Then, the spent medium was replaced and the cells were exposed to a series of doses of free PTX, empty PLGA/Au-HS NPs, PTX-PLGA/Au-HS NPs, and PTX- PLGA/Au-HS-RGD NPs contained in DMEM medium at 37 °C. After 24 h of incubation, the medium was withdrawn and 10 μL of CCK-8 reagent was added to each well; after 2 h, the absorption at 450 nm of the cell samples was measured with a UV–Vis microplate absorbance reader (Bio-Rad model 689, Hercules, CA, USA). DMEM and cisplatin solution were used as negative and positive controls, respectively. The viability percentages of the cells exposed to the NPs were expressed in proportion to the control group. The data are presented as the mean ± standard deviation of triplicate measurements.

### 2.11. Cell Assays of Simultaneous Chemo-Photothermal Effect

PTX-concentration-loaded PLGA/Au-HS-RGD NPs with cell viabilities of ca. 70% were used to examine the efficacy of the simultaneous therapeutic activity of photothermal and chemotherapies. HeLa and MDA-MB-231 cancer cells were seeded into 96-well plates and grown for 24 h in standard culture. Different particle formulations were analyzed: (a) a control formulation with only light irradiation without particles, (b) empty PLGA/Au-HS-RGD NPs with irradiation, (c) PTX-PLGA/Au-HS-RGD NPs, and (d) PTX- PLGA/Au-HS-RGD NPs with irradiation. After 2 and 24 h of incubation, the cells were illuminated with an 808 nm laser at 0.5 W cm^−2^ for 10 min. After 48 h of total incubation, 10 μL of CCK-8 reagent was added to each well, and after 2 h, the absorption at 450 nm of the cell samples was measured as described above. The data are presented as the mean ± standard deviation of triplicate measurements, one-way ANOVAs and Tukey tests were carried out to evaluate statistical differences, and we considered a *p* value of < 0.05 as significant.

## 3. Results

### 3.1. Synthesis and Characterization of PTX-PLGA/Au-HS-RGD NPs

**Figure 1:**
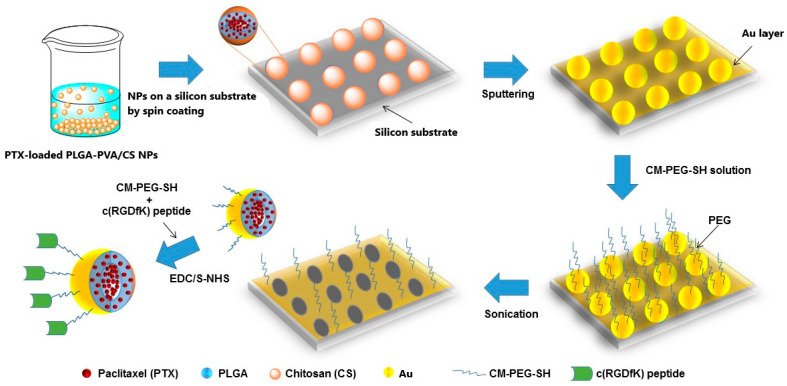
The schematic fabrication process of PTX-PLGA/Au-HS-RGD NPs.

In this study, we developed cyRGD peptide-modified Au half-shell PLGA NPs with specific integrin receptor recognition. Figure 1 shows a schematic representation of the fabrication process. First, PTX-loaded PLGA and empty NPs were prepared using an oil–water simple emulsion. This technique is widely used to incorporate hydrophobic drugs inside amphiphilic polymers such as PLGA [18]. To optimize the amount of PTX loaded into the PLGA NPs, we studied the effects of different PTX amounts (50–100 μg) on the average particle size, PDI, ζ potential, and drug encapsulation. According to the results shown in Table 1, comparing the empty PLGA NPs, PTX loading did not alter, in a significant manner, the average particle size (range from 133 to 144 nm). The PDI of the empty PLGA NPs and different PTX-loaded PLGA NPs was found to be between 0.25 and 0.96, indicating that the NPs prepared had a narrow size distribution, meaning that monodisperse systems were obtained. The ζ potential is an important parameter, as it is commonly an index of colloidal stability. All the formulations obtained exhibited ζ potential values between −20.9 and −25.1 mV due to the carboxyl groups exposed onto the surface of the PLGA NPs. These results confirm that the presence of PTX compared to the empty PLGA NPs did not affect the particle size, PDI, and ζ potential, as reported by other authors [19,20].

The encapsulation efficiency (EE) of the PTX loaded onto the PLGA NPs was determined using the HPLC method. An EE higher than 85% was observed for formulations with 50 to 90 μg of PTX, while for 100 µg, the loss of PTX was high, decreasing the EE to 74% (Table 1). These results agree with previously reported data by Bojat et al., who found that PTX:PLGA ratios below 1:100 ensured a low drug loss [21]. Based on our results, it can be concluded that the formulation obtained using 80 μg of PTX may be considered as optimal for the development of the chemo-photothermal therapy system.

**Figure 2:**
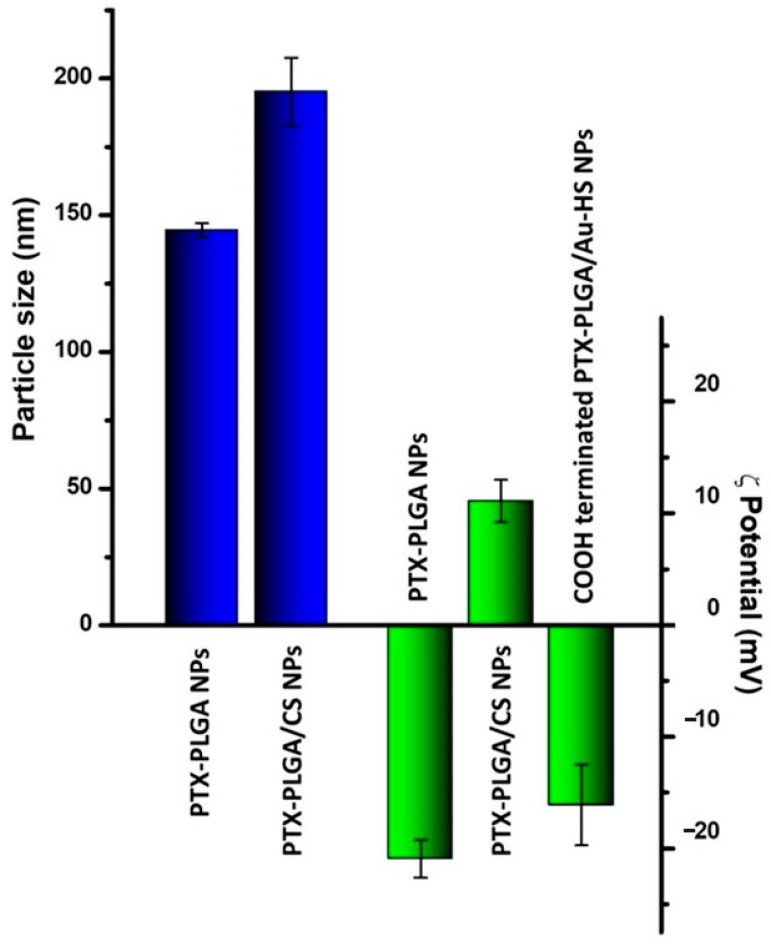
Particle size (blue bar) and ζ potential (green bar) for different stages of the preparation process of PTX-PLGA/Au-HS-RGD NPs.

As is well known, parameters such as particle size, ζ potential, and profile release must be considered in the design and fabrication of drug delivery systems to optimize their stability and biological activity. In this regard, we previously demonstrated how these parameters were tuned in drug-loaded PLGA NPs when a PVA/CS layer was incorporated [16]. Therefore, PTX-PLGA NPs were coated with a CS/PVA layer before being deposited onto silicon substrates. Figure 2 shows the ζ potential and particle size behavior of the PTX-PLGA NPs after the PVA/CS solution incorporation. According to the results obtained, the particle size of the PTX-PLGA-PVA/CS NPs was larger than that of the PTX-PLGA NPs (144.5 ± 2.62 vs. 195.2 ± 12.64 nm). This size increase was due to the CS adsorption through the electrostatic interaction of their positively charged amino groups with negatively charged carboxyl groups onto the surface of the PLGA NPs [22]. Likewise, this effect was observed for the ζ potential, where the adsorbed CS reversed the particle charge of −20.9 ± 1.6 to 11.1 ± 1.9 mV. On the other hand, the PVA presence improved the NPs’ stability; however, due to the formation of hydrogen bonding between their hydroxyl groups and the amino groups of CS, a decreased ζ potential value was obtained [23]. These results suggest that the particle size and ζ potential were adequate for drug delivery system fabrication; in addition, a PVA/CS layer could act as a physical barrier, inducing a slower and sustained released drug, as previously reported [16].

**Figure 3:**
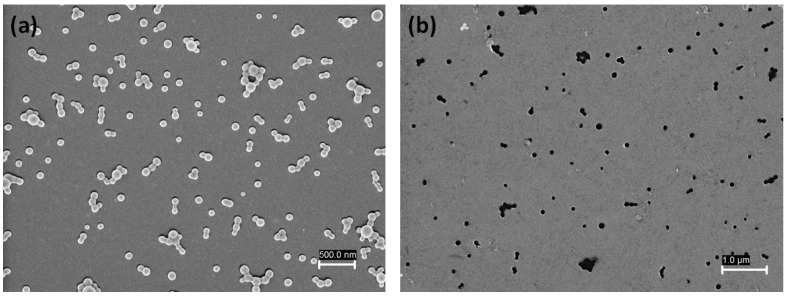
SEM Micrographics of PTX-PLGA-PVA/CS NPs, (**a**) before, and (**b**) after release by sonication.

PTX-PLGA-PVA/CS NPs were deposited onto a silicon substrate via spin coating. To determine the optimal deposition conditions, several substrates with modified parameters such as spinning speed, NP concentration, and volume of solution were studied. Figure 3 shows the SEM micrographs of the PTX-PLGA-PVA/CS NPs deposited onto the silicon substrate before and after the release process via sonication. According to the results of Figure 3a, the NPs exhibited a uniform distribution without signs of aggregation on the substrate surface, an important condition for the development of our system. The NPs presented a spherical shape tendency with a size of ca 98 ± 10 nm, smaller than that determined by DLS, possibly due to the dehydration process resulting from the SEM sample preparation.

**Figure 4:**
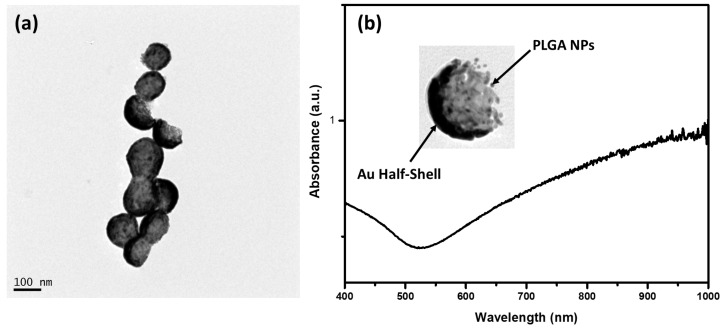
(**a**) TEM micrograph, and (**b**) UV–vis/NIR absorption spectrum of PTX-PLGA/Au-HS-RGD NPs.

In the next step, the substrates were placed inside the COOH-PEG-SH solution so that the PTX-PLGA/Au-HS NPs were released through the sonication process. Thiol-terminated polyethylene glycol (PEG) is commonly used to functionalize the surface of gold nanoparticles. In this regard, the functionalization of the outer faces of the NPs on the substrate with COOH-PEG-SH was carried out via the Au–S bond. Figure 3b shows the SEM micrograph of a substrate after sonication. It can be seen that the presence of small darker dots in the place previously occupied by the PTX-PLGA/Au-HS NPs confirmed their successful release. Next, the COOH-terminated PTX-PLGA/Au-HS NPs were analyzed to confirm the carboxyl group available on the Au half-shell surface. Figure 2 shows the ζ potential results, observing a reversal of the value to −16.1 ± 3.62 mV concerning the PTX-PLGA-PVA/CS NPs, which suggests that the PTX-PLGA/Au-HS NPs presented carboxyl groups on their surface. For targeted delivery purposes, the cyRGDfk peptide with an affinity for the αvβ3 integrins expressed on tumor vascular endothelial cells was conjugated to the carboxyl-group-terminated PTX-PLGA/Au-HS NPs. Figure 4a shows a TEM micrograph of the PTX-PLGA/Au-HS-RGD NPs. The random orientation of the Au half-shells on the substrate could make the morphological characterization difficult. However, half-shell nanostructures with defined polymeric and metal components were clearly observed (inset Figure 4b). Figure 4b depicts the ultraviolet-visible/NIR absorption spectrum of the PTX-PLGA/Au-HS-RGD NPs. A wide absorption peak in the NIR region (from 600 to 1000 nm) was observed, which matches with that previously reported by other authors [8,15,24]. These results suggest that our nanosystem could be successfully exploited for targeted chemo-photothermal therapy.

### 3.2. Photothermal Conversion

**Figure 5:**
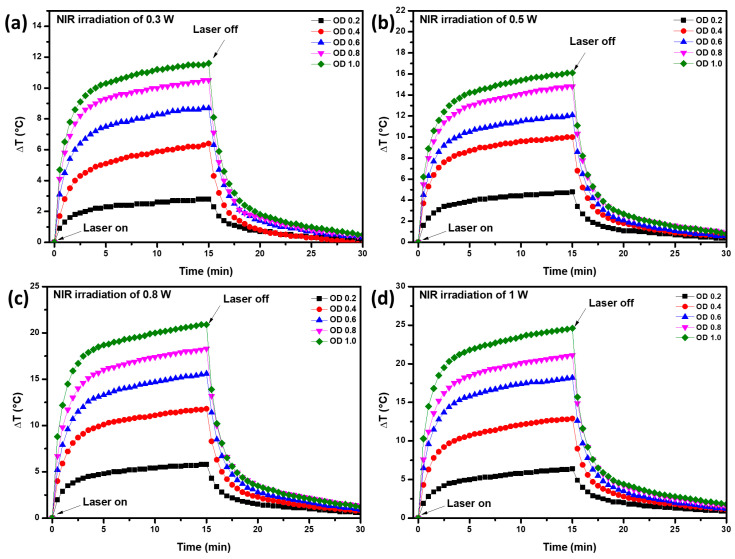
Temperature versus time for the PLGA/Au-HS-RGD NPs with different optical densities and NIR light irradiation for 15 min; (**a**) 0.3 W, (**b**) 0.5 W, (**c**) 0.8 W, and (**d**) 1 W of power.

Figure 5 shows the thermal behavior of the aqueous solutions containing PLGA/Au-HS-RGD NPs, adjusted to different optical densities (0.2, 0.4, 0.6, 0.8, and 1) and irradiated using an NIR laser emitting light at a wavelength of 808 nm with powers of 0.3, 0.5, 0.8, and 1 W cm^−2^. All the experiments exhibited a similar trend after the laser was turned on. The temperature increased rapidly during the first 3 min and then slowly until the laser was turned off (15 min). The ΔT_max_ achieved in our study was 24.6 °C, which corresponds to an optical density of 1, laser power of 1 Wcm^−2^, and beam spot with a diameter of 0.5 cm (Figure 5d), while the ΔT_min_ achieved was 2.8 °C, which corresponds to an optical density of 0.2, laser power of W cm^−2^, and the same beam spot diameter (Figure 5a). It is clear that the ΔT achieved depended on the optical density of the solution and the laser power used during the test. In this process, the ΔT_max_ is considered the ability to convert photons into heat, which depended on the nanoparticle’s absorption efficiency [25]. Lindley et al. synthesized hollow gold nanostructures with photothermal properties and reported increases of 6.5 and 12 °C for samples adjusted at optical densities of 0.2 and 0.5, illuminated by a 790 nm CW laser with a 7 mm spot size and 1.0 W cm^−2^ power density [26]. In our work, higher temperature increments were observed for some evaluated conditions, e.g., for an optical density of 0.6 and laser power of 0.8 W cm^−2^, and temperature increases of 15.6 °C were observed (Figure 5c). On the other hand, Kim et al. performed a photothermal study on a similar gold half-shell structure to ours and observed a ΔT_max_ of 28 °C using a laser power of 0.62 W/cm^2^ and a beam diameter of 3.5 cm. Their ΔT_max_ was similar to that reported in our study, however, they used different experimental conditions [24]. These results suggest that PLGA/Au-HS-RGD NPs are efficient light-to-heat converters, comparable with the reported results for gold nanorods [27,28]

### 3.3. In Vitro PTX Release

**Figure 6:**
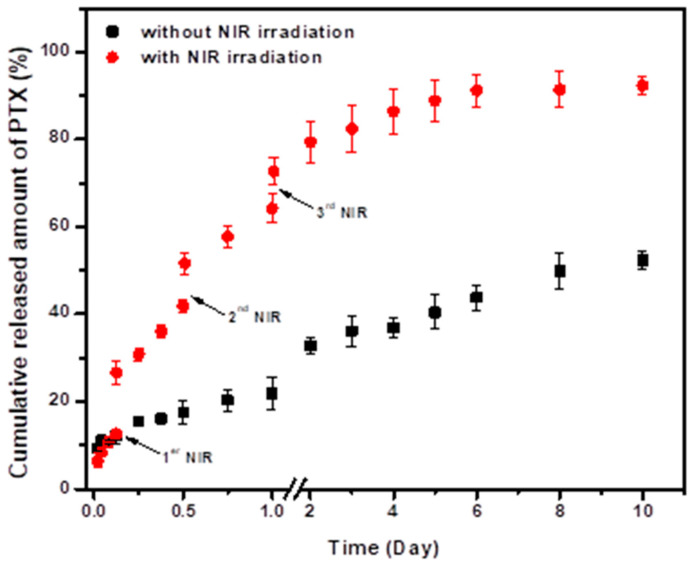
Profiles of PTX release from PTX-PLGA/Au-HS-RGD NPs with and without NIR irradiation of 0.5 W cm^−2^ for 5 min at the 3, 12, and 24 h after the start of experiment. Data represent mean values for *n* = 3 and the error bars represent standard deviation of the means.

The release profiles of the PTX from the PLGA/Au-HS-RGD NPs with and without NIR irradiation are shown in Figure 6, and a sustained PTX release for a period as long as 10 days can be observed in both conditions, reaching values of about 92% and 49% for the NPs with and without laser irradiation. The burst release was very low in both cases, and this fact is very valuable for drug delivery systems that are pretended to be used to treat solid tumors, due to the fact that e cargo molecules should not be released into the bloodstream, assuring that drugs can reach tumor sites at high concentrations, increasing their efficiency and diminishing side effects. Sakhi et al. encapsulated paclitaxel in PLGA nanoparticles, and the best conditions synthesis showed a burst release of around 30% of the paclitaxel in the first 24 h [29]; in our work, 20% of the drug was released at the same time in the absence of laser irradiation.

On the other hand, it was clearly shown that laser irradiation potentiated the PTX release, as a significant increase in the drug released was observed each time that the NPs were subjected to it. At the end of the experiment, almost all the encapsulated PTX was released from the nanoparticles after the NIR irradiation. Temperature increases induced by NIR irradiation could promote drug diffusion, potentiating release from nanoparticles; in addition, the PLGA degradation rate was higher as the temperature increased [30]. Furthermore, it has been reported that PLGA nanoparticles exhibit a glass transition temperature (Tg) at 43–46 °C [31]. The Tg is an important parameter for semi-crystalline and amorphous polymers, as when glass transition take place, the chains in the amorphous portion can escape their entanglements, which increases the polymer mobility and can promote PTX release from nanoparticles [32,33].

### 3.4. Cell-Association and Internalization of PLGA/Au-HS-RGD NPs

**Figure 7:**
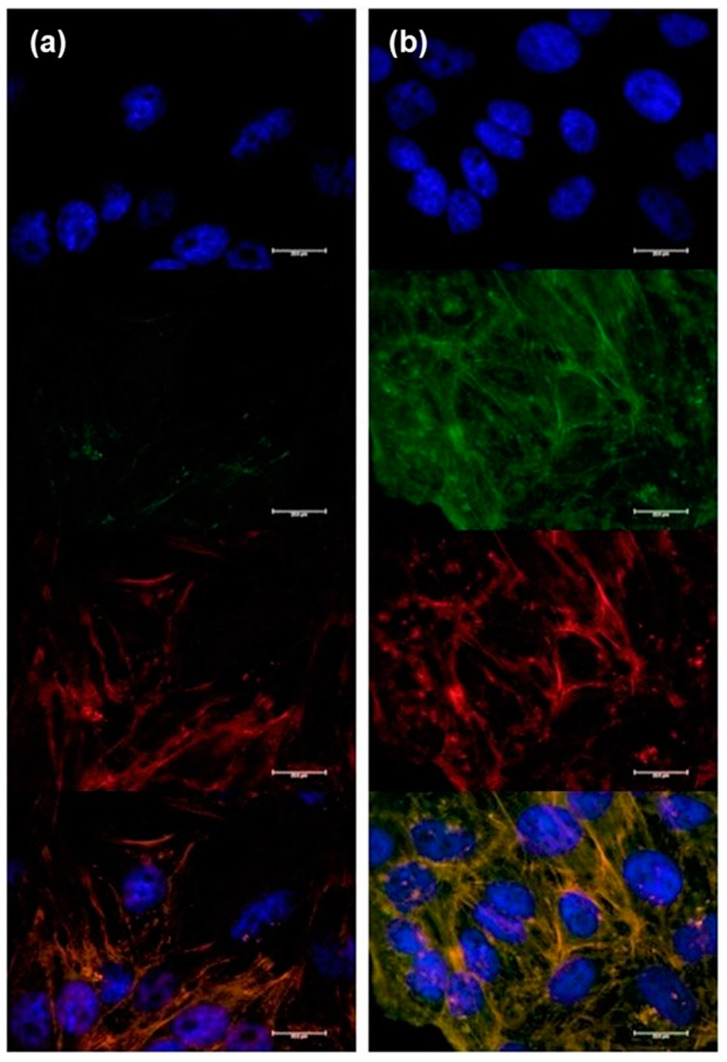
Confocal microscopy images of HeLa cells. Cells nuclei were stained with DAPI, NPs were stained with FITC. (**a**) HeLa cell exposed to PLGA/Au-HS NPs for 4 h. (**b**) HeLa cell exposed to PLGA/Au-HS-RGD NPs for 4 h. Scale bar 20 μm.

Gold nanoparticles with cyRGDfk (RGD) surface functionalization have been extensively studied for their potential in diverse biomedical applications, such as drug delivery, imaging, and cancer therapy [34]. One of the critical aspects of peptide-grafted NPs surfaces is their ability to internalize into target cells, which is crucial for enhancing their therapeutic efficacy. HeLa cells, for investigating the cellular internalization of NPs, were exposed to PLGA/Au-HS-RGD NPs and PLGA/Au-HS NPs loaded with FTIC to observe the peptide effect on the cellular uptake. As the confocal microscopy images showed (Figure 7), NPs modified with RGD peptide increased the cellular uptake of cells compared to the negative control (within RGD). This observation was due to specific receptor-targeting peptides such as RGD presenting a high affinity to their homologous receptors, ανβ3 y ανβ5 integrins. The internalization of PLGA/Au-HS NPs with RGD surface functionalization has been demonstrated in various in vitro and in vivo studies. For instance, studies have shown that these NPs can efficiently internalize into cancer cells and accumulate in tumor tissues in vivo [35,36,37]. As shown in Figure 7b, detectable green fluorescence was observed in the cell cytoplasm of the HeLa cells after 4 h of incubation after fluorescent NPs administration.

The current findings are consistent with previous research that has used integrin receptors as specific targets to assist with the therapeutic action of other bioactive chemicals, such as IR780 NIR dye encapsulated in lipid nanoparticles for HEK29(3)-vRFP cells [38], anti-STAT3 siRNA for B16F10 melanoma cells using cationic liposomes [39], and DSPE-PEG micelles to encapsulate indocyanine-green-targeting SGC7901 gastric cancer cells [40] among others. Albertini et al. [41] reported nanotechnology approaches for cancer diagnosis and treatment based on utilizing AuNPs coated with c-RGD peptide. The accumulation of peptide-decorated NPs in the subcutaneous tumor of mice two hours after administration was 4-fold higher than that of uncoated particles and 1.4-fold higher than that of PEGylated particles, demonstrating the high potential of surface-modified peptide carriers for the diagnostic and therapeutic delivery of PTX applications.

Integrin receptors that are overexpressed on the surface of cancer cells can be specifically targeted thanks to the c-RGD functionalization of the NP surface. The peptide c-RGD interacts with the integrins αvβ3 and αvβ5, which are implicated in angiogenesis, tumor development, and metastasis [42]. Overall, the internalization of RGD-functionalized PLGA/Au-HS NPs is a promising strategy for targeted drug delivery and imaging in cancer therapy. Figure 7b shows that NPs are internalized into the cell cytoplasm, observing green fluorescence from FITC overlaid with the bright field cytoplasm location, with no apparent fluorescence within the nucleus. Once internalized, NPs might successfully achieve a sustained release via the PLGA hydrolysis of their cargo into the cells, enhancing the PTX therapeutic activity.

### 3.5. Cell Viability

**Figure 8:**
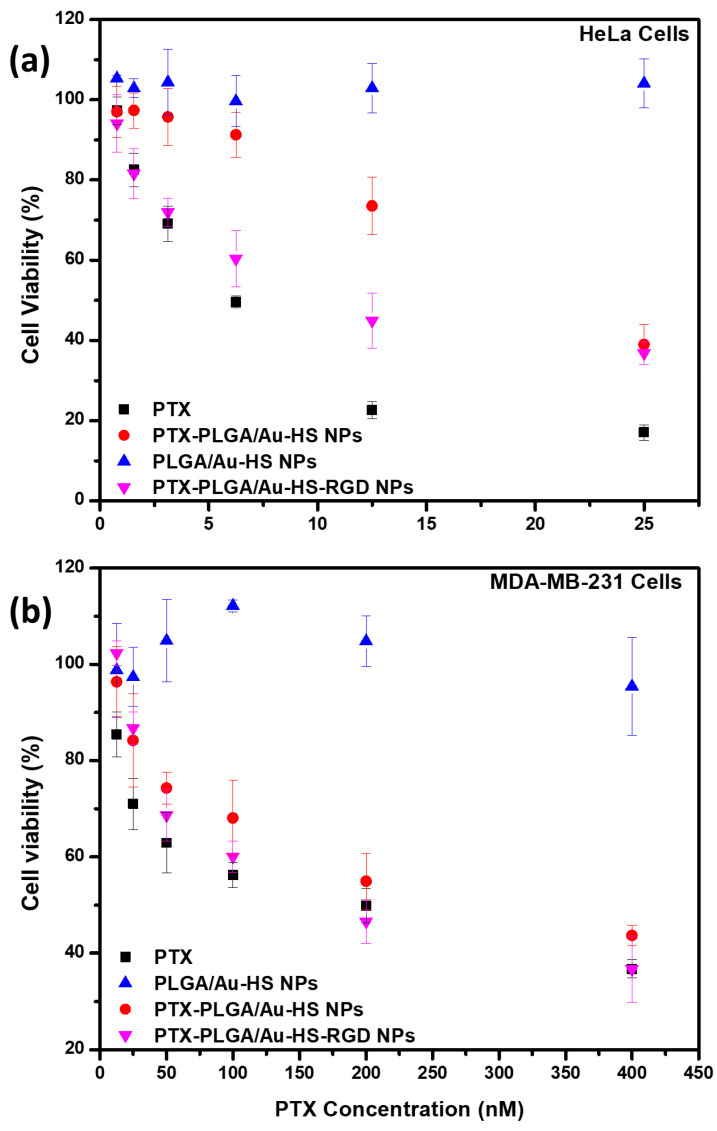
In vitro cytotoxicity of free PTX, empty PLGA/Au-HS NPs, PTX-PLGA/Au-HS NPs, and PTX-PLGA/Au-HS-RGD NPs; (**a**) HeLa cells, and (**b**) MDA-MB-231 cells.

In vitro cytotoxicity evaluation experiments using HeLa and MDA-MB-231 cells, which overexpress integrin αvβ3 and αvβ5 receptors at cell membrane surfaces, were performed to evaluate the therapeutic efficacy of combined PTX-PLGA/Au-HS NPs with RGD peptide (Figure 8).

It can be observed that, when immobilized RGD is present, a reduced amount of encapsulated PTX is needed to reduce, at 50%, cell viability. Figure 8 shows the cell viabilities at different PTX concentrations (0.25–25 nM) after 48 h. For the two lowest cargo concentrations at 48 h, the treatment significantly reducing the HeLa cell proliferation was raw PTX and PTX-PLGA/Au-HS-RGD NPs, ca. 20%, compared to ≈1% for the PLGA/Au-HS NPs. These observations were consistent for the MDA-MB-231 cells, where PTX-PLGA/Au-HS-RGD NPs reduced the cell viability to a greater extent at lower PTX concentrations. The IC50 values for the treatments were 6.93 nM, 20 nM, and 10 nM for the PTX, PTX-PLGA/Au-HS NPs, and PTX-PLGA/Au-HS-RGD NPs against the HeLa cell line. Conversely, the IC50 values against the MDA-MB-231 cells resulted to be 212.89 nM, 265 nM, and 201 nM for the PTX, PLGA/Au-HS NPs, and PTX-PLGA/Au-HS-RGD NPs, respectively. Cancer patients are regularly prescribed the chemotherapeutic medication PTX. Unfortunately, breast cancer resistance to PTX treatment is a significant barrier in clinical applications and one of the leading reasons for death in patients who fail to respond to treatment [43]. Due to its poor solubility and off-target effects, however, its efficacy may be constrained. These issues can be resolved and the drug’s therapeutic efficacy through sustained release can be increased by encapsulating PTX in PLGA/Au-HS-RGD NPs.

Integrin receptors are attractive targets for cancer therapy, and targeting them using peptides has been shown to improve the specificity and efficacy of cancer treatments. Similar results have been reported by many researchers using RGD-peptide-targeting integrin receptors. Cao et al. [44] studied the potential of RGD-PTX-liposomes for targeting tumor cells and enhancing the therapeutic effect of PTX. The RGD-PTX-liposomes were found to have a greater affinity for tumor cells than the non-modified PTX-liposomes, highlighting their potential for targeted drug administration. The RGD-PTX-liposomes also had a stronger anti-tumor impact in vitro and in vivo than the non-modified PTX-liposomes. Because of their precise targeting of cancer cells by binding to the integrin αvβ3 and αvβ5 receptors that are overexpressed on the surface of many cancer cells, similar to our results observed for the HeLa cells, the RGD-targeted vectors were found to be more cytotoxic and had a stronger therapeutic effect than the non-targeted vectors. Non-targeted vectors can bind to and enter both cancer and normal cells, resulting in off-target effects and damage to healthy cells. These observations can reduce antineoplastic drugs’ therapeutic impact while also causing undesired side effects. RGD-targeted vectors, on the other hand, can selectively bind and enter cancer cells that overexpress integrin v3 receptors, decreasing their contact with healthy cells and therefore their cytotoxicity.

Nanoparticles can be engineered to specifically target cancer cells while minimizing damage to healthy cells, which reduces the risk of side effects associated with traditional cancer therapies. Peptides can bind to specific receptors on the surface of cancer cells, increasing the nanoparticles’ specificity for the cancer cells and minimizing the binding to healthy cells. Integrin-targeting peptides can be attached to the surfaces of nanoparticles, which can selectively bind to the integrin receptors on cancer cells. The binding of the peptide to the integrin receptor can trigger the internalization of the peptide–nanoparticle complex into the cancer cell, leading to an increased uptake and higher concentrations of therapeutic agents within the cancer cells. In addition to improving drug delivery, integrin-targeting peptides can also inhibit the activity of integrin receptors, which can reduce cancer cell migration and invasion. For example, cyclic RGD peptides have been shown to inhibit tumor growth and metastasis by blocking the activity of αvβ3 integrin receptors [45,46]. Attaching peptides to nanoparticles can improve cancer treatment targeting and therapeutic efficacy, while reducing the risk of side effects.

### 3.6. In Vitro Cellular Cytotoxicity Assays and Photothermal Test

**Figure 9:**
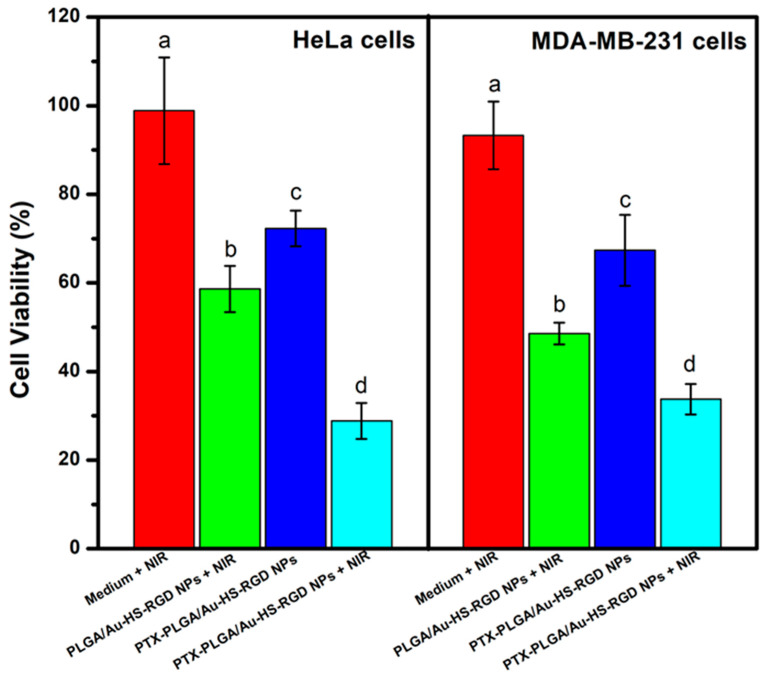
Viability of HeLa and MDA-MB-231 cells with chemo-photothermal therapy. Hela and MDA-MB-231 were treated with PTX-free PLGA-Au-HS, PTX- PLGA/Au-HS NPs, and PTX-PLGA/Au-HS-RGD NPs with NIR irradiation. Different letters indicate significant differences (*p* < 0.05).

In preclinical studies, combining photothermal therapy with chemotherapy utilizing gold nanoparticles and paclitaxel has shown excellent results. The main idea is to use gold nanoparticles to selectively increase the temperature in tumor cells and the surrounding environment, making them more vulnerable to chemotherapeutic effects. Figure 9 shows the effect of combining therapies after laser ablation using laser and PTX. For the in vitro assay, the HeLa and MDA-MB-231 cell lines were exposed to PTX-PLGA/Au-HS-RGD NPs with PTX concentrations of 3.125 and 50 nM, respectively. After the treatment with the PTX-PLGA/Au-HS-RGD NPs, the cell viability was significantly reduced to 72% in the HeLa cells and 67% in the MDA-MB-231 cells compared to the control. Next, the treatment with the PTX-PLGA/Au-HS-RGD NPs exposed to NIR light (0.5 W/cm^−2^) using a laser diode (λ = 808 nm) was performed at 2 and 24 h after the start of the experiment for 10 min, followed by further incubation at 37 °C. Upon NIR irradiation, the temperature increased to approximately 45 °C, comparable to conventional hyperthermal treatments’ temperature. In this case, the cell viability was reduced to 23% for the HeLa cells and 31% for the MDA-MB-231 cells. The combination of PTX with NIR irradiation significantly enhanced the cytotoxic effect on the HeLa (*p* = 0.00033) and MDA-MB-231 (*p* = 0.00053) cell lines as a result of the plasmonic heating of the PTX-PLGA/Au-HS-RGD NPs.

It is crucial to highlight that the NIR heating of PLGA/Au-HS NPs for in vivo photothermal therapy may necessitate the active targeting for certain malignancies to be most effective, as adequate collective photothermal conversion is required to achieve the necessary cell hyperthermic temperatures. NIR penetration to the tumor location is also required for effective treatment.

The functionalization of PLGA/Au-HS NPs with RGD peptide has been proposed as a tumor-cell-targeting approach. When RGD-functionalized gold nanoshells are administered, they specifically connect to the integrin receptors on the surface of tumor cells, causing them to accumulate in the tumor tissue. PLGA/Au-HS-RGD NPs can be activated by near-infrared (NIR) light once they have been localized in this tumor tissue. Furthermore, when activated with NIR light, the localized heating generated by the NPs can selectively eliminate tumor cells without damaging the surrounding healthy tissues, significantly decreasing adverse effects. Different Au nanoparticles have been used to demonstrate the importance of functionalizing NPs with RGD peptide. Li et al. [47] showed that polydopamine-coated Au nanostars could specifically bind to HCC cells, and upon activation with near-infrared (NIR) light, induce the selective photothermal ablation of the tumor cells. In a mouse model of HCC, the authors found that this strategy significantly reduced tumor development while causing no discernible adverse effects. The study also compared the effectiveness of integrin-αvβ3-targeting peptides with non-targeted nanoparticles and discovered that the latter had a substantially higher concentration in the tumor tissue and a more substantial therapeutic effect.

Overall, the results comprehensively assessed the efficacy and safety of combining PTT and chemotherapy approaches for cancer treatment. The observations suggest that using integrin-αvβ3-targeting peptides to functionalize PLGA/Au-HS NPs may be a promising strategy for the targeted delivery of therapeutic agents to in vivo systems, while sustaining the delivery of PTX mediated by PLGA.

## 4. Conclusions

In the current study, we synthesized a peptide-functionalized stimuli-responsive paclitaxel delivery system with a high sensitivity to NIR irradiation. The system showed a sustained release for as long as 10 days and an excellent response to laser irradiation, attaining the release of almost all the PTX encapsulated in the nanoparticles when the stimulus was applied. Furthermore, we demonstrated how photothermal therapy and PTX- PLGA/Au-HS-RGD NPs exhibit synergistic therapeutic advantages in cancer treatment in vitro. When combined with NIR light, these formulations demonstrated about a 40% reduced cell viability against HeLa and MDA-MB-231 cell lines compared to the same nanoparticles without irradiation. Additionally, we showed that RGD peptide was a reliable molecule to concentrate our system in cells overexpressing integrin receptors due to selective targeting; this could help patients require lower PTX doses while lowering side effects. These findings suggest that targeted chemo-photothermal therapy from PTX-PLGA/Au-HS-RGD NPs can be a potential alternative for solid tumor treatment. However, future work should be concerned with in vivo evaluations using animal models that allow for the designing of new cancer therapy programs. The biocompatibility and safety profile of gold half-shell coated PLGA nanoparticles should be addressed. Extensive in vivo toxicity studies should be conducted to assess their long-term impacts, biodistribution, and potential accumulation. Investigating techniques for preventing adverse effects and optimizing the formulation to reduce off-target effects will also be critical for developing effective and safe nanotherapeutic platforms for cancer treatment, bringing us closer to personalized and precise cancer therapy. Investigating ways to limit undesirable effects and refining the formulation to reduce off-target effects will also be crucial for developing effective and safe nanotherapeutic platforms for cancer treatment, bringing us closer to customized and precise cancer therapy.

## Figures and Tables

**Table 1:** Encapsulation efficiency, average particle size, ζ potential, and polydispersity index of empty PLGA NPs and PTX-loaded PLGA NPs (*n* = 3, mean ± standard deviation).

Initial PTX (μg)	PTX Encapsulation (%)	Particle Size (nm)	ζ Potential (mV)	PDI
--	--	133 ± 3.9	−23.5 ± 2.5	0.062
50	90.4 ± 3.6	138 ± 6.7	−25.1 ± 1.7	0.025
60	85.7 ± 7.5	139.4 ± 5.4	−23.4 ± 0.2	0.044
70	86.2 ± 7.7	133.5 ± 2.6	−21.9 ± 1.8	0.078
80	93.9 ± 2.3	144.6 ± 2.6	−20.9 ± 1.7	0.078
90	86 ± 8.1	142.2 ± 9.1	−24.2 ± 3.06	0.096
100	74.5 ± 9.9	139.8 ± 4.8	−24.3 ± 0.9	0.086

## Data Availability

Data sharing not applicable.
